# Research on Online Monitoring Technology and Filtration Process of Inclusions in Aluminum Melt

**DOI:** 10.3390/s24092757

**Published:** 2024-04-26

**Authors:** Yunfei Wu, Hao Yan, Jiahao Wang, Jincan Zheng, Xianzhao Na, Xiaodong Wang

**Affiliations:** 1 State Key Laboratory of Advanced Steel Processes and Products, Central Iron and Steel Research Institute, Beijing 100081, China; yfwu0018@foxmail.com; 2 College of Materials Science and Opto-Electronic Technology, University of Chinese Academy of Sciences, Beijing 100049, China; yanhao211@mails.ucas.ac.cn (H.Y.); wangjiahao21@mails.ucas.ac.cn (J.W.); zjc_2009@sina.com (J.Z.)

**Keywords:** aluminum melt, online monitoring, inclusions, signal processing, aluminum alloy filtration process

## Abstract

Online monitoring and real-time feedback on inclusions in molten metal are essential for metal quality control. However, existing methods for detecting aluminum melt inclusions face challenges, including interference, prolonged processing times, and latency. This paper presents the design and development of an online monitoring system for molten metal inclusions. Initially, the system facilitates real-time adjustment of signal acquisition parameters through a multiplexer. Subsequently, it employs a detection algorithm capable of swiftly extracting pulse peaks, with this task integrated into our proprietary host computer software to ensure timely detection and data visualization. Ultimately, we developed a monitoring device integrated with this online monitoring system, enabling the online monitoring of the aluminum alloy filtration process. Our findings indicate that the system can accurately measure the size and concentration of inclusions during the filtration process in real time, offering enhanced detection speed and stability compared to the industrial LiMCA CM (liquid metal cleanliness analyzer continuous monitoring) standard. Furthermore, our evaluation of the filtration process demonstrates that the effectiveness of filtration significantly improves with the increase in inclusion sizes, and the synergistic effect of combining CFF (ceramic foam filter) and MCF (metallics cartridge filter) filtration methods exceeds the performance of the CFF method alone. This system thus provides valuable technical support for optimizing filtration processes and controlling inclusion quality.

## 1. Introduction

Non-metallic inclusions significantly impact the mechanical properties of metals, undermining their ductility, tensile strength, fatigue strength, and corrosion resistance, consequently degrading the quality of the final product [1]. The origins of aluminum inclusions are predominantly oxide formations from oxidation reactions, external nucleating agents, slagging on the furnace wall during melting, and erosion of refractory materials [2,3]. These inclusions are primary contributors to ductile dimple and fatigue cracks, with an increase in inclusion size further diminishing alloy strength [4,5,6]. Research indicates that the corrosion resistance and ductility of aluminum alloys suffer directly from the volume of inclusions present [7,8,9]; a surge in inclusion content notably reduces these properties in aluminum alloy materials [10]. Studies, including Spriestersbach’s work utilizing ultrasonic tensile compression fatigue tests, have established that the size and type of inclusions crucially affect the fatigue limit of high-strength steel under ultra-high cyclic fatigue conditions [11].

In the aluminum casting process, degassing and filtration represent critical steps for inclusion removal. The typical procedure incorporates spinning nozzle inert flotation (SNIF), ceramic foam filter (CFF), and metallics cartridge filter (MCF). SNIF employs a gas blowing approach, introducing a blend of inert and chlorine gases into the melt via a rotor, which then disperses small bubbles evenly throughout the aluminum melt. These bubbles ascend, absorbing hydrogen and entrapping inclusions, thus purifying the aluminum melt. The injection of chlorine gas facilitates the formation of chlorides with alkali metals, which are expelled as the gas ascends [12,13,14]. Following degassing, CFF serves as the primary filtration stage, utilizing adsorption filtration. Here, the aluminum melt encounters the foam ceramic filter plate, where impurities are trapped in the plate’s pores and cavities due to sedimentation and inertia. This method effectively separates slag from liquid, excelling in the removal of large inclusions [15,16,17]. As a secondary filtration measure, MCF mirrors CFF’s mechanism but boasts a greater filtration area. CFF’s prior removal of large inclusions prevents “cake layer” formation on the MCF filter tube, enhancing its filtration efficiency. MCF’s finer filtration precision capitalizes on the deep filtration capability of the filter tube, optimizing filtration efficacy [18].

The commonly utilized methods for detecting inclusions currently encompass the metallographic method, PoDFA (porous disc filtration analyzer) (Asea Brown Boveri, Zurich, Switzerland), LAIS (liquid aluminum inclusion sampler) (Union Carbide Corporation, Danbury, CT, USA), and the K-mold method. These methodologies offer the advantage of simplicity in operation. However, they are all offline measurements, which inherently results in a delay in outcomes, thereby impeding real-time monitoring and control of the molten metal’s cleanliness level. Additionally, offline sampling may alter the composition and size distribution of inclusions [19,20,21]. Evaluating the removal efficiency of inclusions during the aluminum liquid filtration process, alongside real-time online monitoring of inclusion size and distribution, is critically important for maintaining and enhancing the quality of aluminum products. Presently, two theoretical approaches facilitate real-time online measurement of inclusion content in molten metal: the ultrasonic method and the electrical sensing zone method. The sensitivity limit of ultrasonic testing correlates with the frequency of the ultrasound; a higher frequency allows for the detection of smaller inclusions, albeit with increased ultrasonic energy attenuation, which imposes a limit on the detection frequency [22,23]. The minimum inclusion size detectable by ultrasonic methods is currently only 60 μm. In the 1950s, Coulter introduced the electrical sensing zone method, establishing a Coulter counter based on this method to address red blood cell measurement challenges [24]. Coulter counters offer a more convenient, efficient, and precise alternative to traditional red blood cell measurement techniques. In 1985, McGill University adapted the electrical sensing zone method for metallurgical applications, naming it LiMCA (liquid metal cleanliness analyzer) (Asea Brown Boveri, Zurich, Switzerland) technology [25]. ABB (Asea Brown Boveri) has since commercialized LiMCA technology for assessing liquid metal cleanliness in the metallurgical industry, leading to the development of a range of commercial LiMCA products [26,27].

In response to the technical challenges and immediate needs associated with the online monitoring of inclusions in aluminum melt, this paper presents the design of an online monitoring system. This system is capable of real-time adjustments to signal gain and filter passbands and incorporates a novel peak detection algorithm within its software to guarantee the promptness of both detection and data visualization processes. The bespoke online monitoring system excels in cost-effectiveness, speed, accuracy, and the efficiency of its pulse peak detection algorithm, thereby extending the detection range for inclusions. Building on the system’s design, a monitoring device equipped with this technology was constructed. Utilizing this device, real-time, online, and direct measurements were conducted on aluminum melt subjected to SNIF, CFF, and MCF processes during the aluminum alloy casting procedure. These measurements facilitated the assessment of the filtration efficacy across different processes. The findings indicate a significant reduction in both size and concentration of inclusions within the filtered aluminum melt, with the impact more pronounced for larger inclusions. Moreover, the combined filtration effect of CFF + MCF surpasses that of CFF alone. This system overcomes the traditional online monitoring methods’ limitation of static sampling parameters by incorporating dynamic adjustment capabilities and integrating the peak detection algorithm directly into the host computer software, thereby lowering the cost of online inclusion monitoring. It uncovers the effects of the filtration process on the distribution and variability of inclusions, laying the groundwork for future enhancements in filtration techniques and inclusion quality control. Furthermore, it introduces an innovative approach to the online detection of inclusions in various melts, including aluminum, zinc, and steel.

## 2. The Basic Principle of Detecting Non-Metallic Inclusions in Aluminum Liquids and Online Monitoring System

### 2.1. Electrical Sensing Zone Method

**Figure 1:**
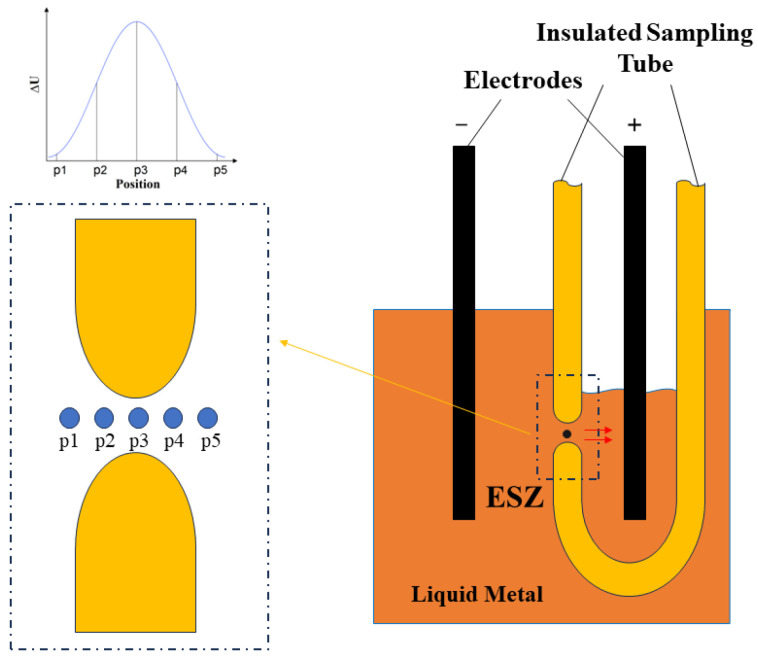
Schematic diagram of the principle of the electrical sensing zone method, (The red arrow is the direction of molten metal flow).

The principle of the electrical sensing zone method is depicted in Figure 1. This sensor comprises an insulated sampling tube equipped with a diminutive orifice on its sidewall, inside which positive and negative electrodes are mounted to maintain a constant current. During measurement, a negative pressure is applied to the tube, causing inclusions in the molten metal to enter alongside the liquid metal through these small orifices, resulting in a resistance change within. This change, under the constant current’s influence, leads to alterations in the potential across the electrodes. Given that such potential shifts occur exclusively within the tiny orifice, this region is designated as the electrical sensing zone (ESZ). The passage of a particle through the ESZ generates a potential change, translating into a voltage pulse signal that is rich in particle information: the pulse’s height reflects the particle’s diameter, its width indicates the velocity at which the particle traverses the ESZ, and the pulse count aligns with the quantity of particles passing through the ESZ in the molten metal. Consequently, this technique is characterized by its foundational superiority and exceptional measurement precision. The correlation between voltage pulse amplitude and particle size is delineated by the subsequent equation [25]:

(1) $$∆U=\frac{4\rho_{m}d^{3}}{\pi D^{4}}I$$

where ∆U is the peak voltage pulse; $\rho_{m}$ is the resistivity of the liquid metal; d is the nominal diameter of the particles; D is the diameter of the side orifice; and I is the constant current.

### 2.2. Non-Metallic Inclusions Online Monitoring System

**Figure 2:**
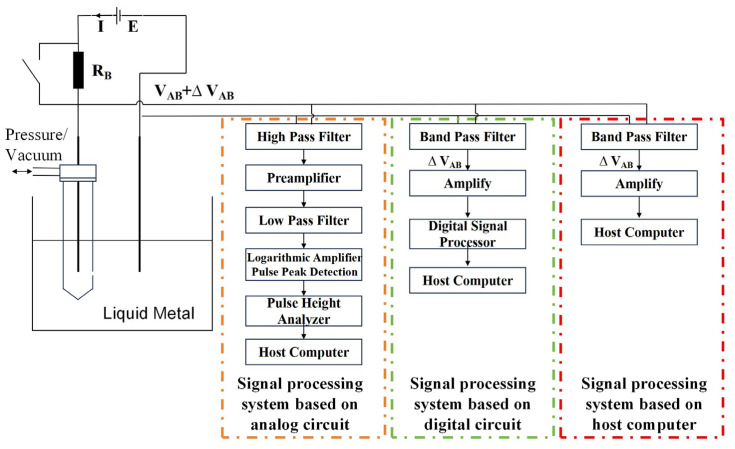
Schematic diagram of non-metallic inclusions online monitoring system.

There are two primary signal processing methodologies for monitoring systems based on the ESZ method [28], as illustrated in Figure 2. The first is reliant on analog circuits, predominantly utilized in LiMCA I, where the pulse signal undergoes sequential amplification and filtering through an operational amplifier and filter, subsequently being directed to a logarithmic amplifier and peak detector for voltage pulse detection. The extraction of pulse information is performed by a pulse height analyzer before the data are relayed to the host computer. With the advent of digital circuit technology, LiMCA II transitioned from analog to digital circuits, a change adopted by LiMCA II and later versions in their signal processing modules. Within these modules, peak detection and pulse height analysis are executed by a digital signal processor. The shift to digital circuits has allowed LiMCA products to substantially decrease in size and cost, facilitating the portability of LiMCA measurements.

In light of the swift advancements in software technology in recent years, this study has conceptualized and constructed an online monitoring system for inclusions that leverages host computer software. This system’s host computer software encompasses data display functionalities and manages signal post-processing activities, including pulse peak extraction, height analysis, and pulse–particle size conversion. The pulse signal is first filtered through a bandpass filter to eliminate noise and then amplified prior to being directly forwarded to the host computer. Subsequently, the host computer employs a peak detection algorithm to identify the peak value of the inclusion pulse and utilizes Equation (1) to convert it into the inclusion’s size.

**Figure 3:**
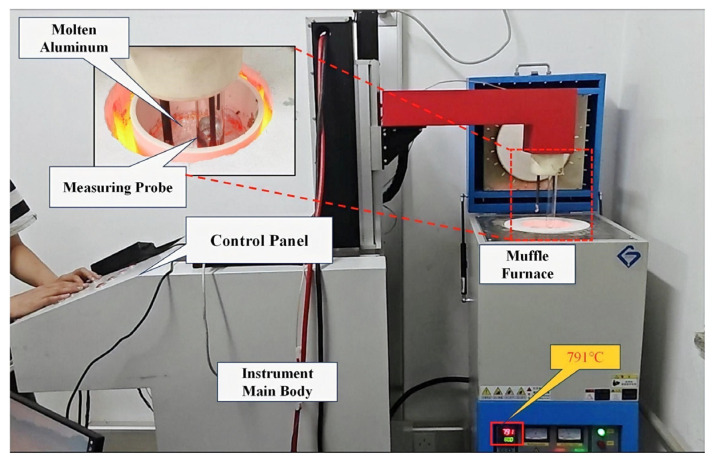
Experimental measuring setup.

Figure 3 illustrates a measurement device equipped with a bespoke online monitoring system for detecting inclusions. At the experiment’s onset, the device employs a mechanical arm, maneuvered via the control panel, to insert the sampling probe into the crucible, thereby submerging the sampling tube and electrode into the crucible’s aluminum melt. Negative pressure is then applied within the sampling tube, facilitating the entry of the aluminum melt. Throughout the experiment, the aluminum melt is cyclically extracted by alternating positive and negative pressures in the sampling tube, enabling the quantification of inclusions traversing the ESZ. The device solely tallies the inclusions during the aluminum melt’s intake phase. Although the discharge phase’s detection signal of the aluminum melt is visualized on a real-time waveform, it does not contribute to the histogram representing particle size distribution.

The focus of this study is the three series alloy from a recycling aluminum casting facility in Shandong. Online measurements were performed under controlled laboratory conditions over a set duration, utilizing a sampling tube designed with a 500 μm orifice. The current was maintained at a constant 50 A. This study introduces the metric N20 to meet the specific requirements of industrial applications, defining it as the count of inclusions larger than 20 μm per kilogram of aluminum melt, expressed in thousands per kilogram (k/kg).

## 3. Design and Development of Signal Processing and Feature Extraction Algorithms

### 3.1. Design and Optimization of Signal Acquisition Module

**Figure 4:**
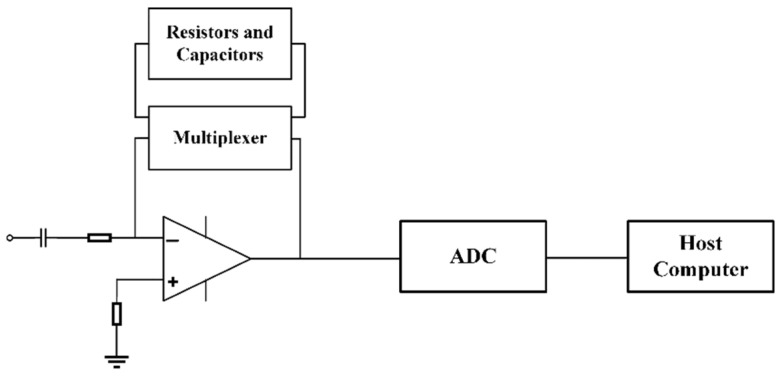
Schematic diagram of the signal acquisition module.

The signal acquisition module plays a pivotal role in the online monitoring system for detecting inclusions, setting the detection threshold for the smallest discernible inclusions. As inclusions traverse the sampling orifice in aluminum melt, they generate analog voltage pulse signals. These signals are then amplified and refined by active filters, subsequently being converted to digital form through analog-to-digital converter. Within the LiMCA product’s signal processing module, signal amplification is consistently set to a 1000-fold increase, with the bandpass filter’s frequency range fixed between 0.1 kHz and 10 kHz, enabling the detection of inclusions larger than 20 μm. Given the challenging conditions prevalent at metal-casting facilities, where noise interference differs across various metals and processes, a rigid amplification and fixed filter frequency range may not represent the most effective design for signal acquisition modules. This research achieved real-time modification of signal amplification and cutoff frequency by incorporating a multiplexer, as depicted in Figure 4. The pulse signal, conveyed to the circuit board via a signal line, sequentially traverses a capacitor and an active filtering circuit. Capacitors serve to eliminate direct current elements and low-frequency noise from the signals, significantly mitigating signal baseline fluctuation effects. The active filtering circuit both amplifies and filters the signal, with its amplification and cutoff frequency being determined by the resistance and capacitance values within the feedback loop. Unlike traditional designs, this study’s signal acquisition module connects the feedback loop of the active filtering circuit not to fixed resistors and capacitors but to those with variable parameters through a multiplexer. During experimental procedures, operators can adjust the multiplexer’s parameters via the host computer software, altering the signal amplification and filter’s frequency range accordingly.

### 3.2. Optimization of Pulse Peak Detection Algorithm Based on Software Technology

**Figure 5:**
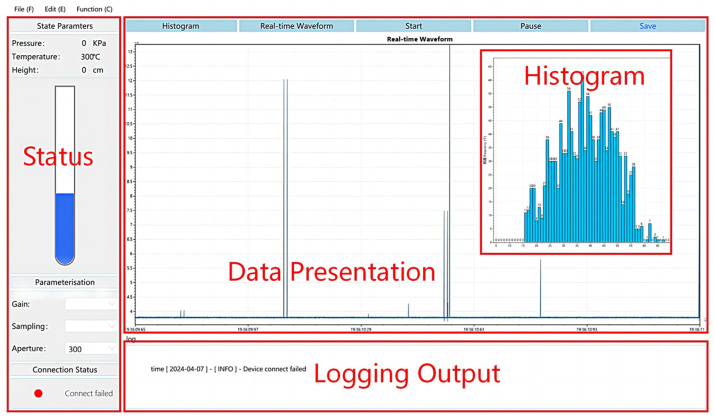
The software interface and functional area division of the host computer.

In the inaugural series of LiMCA products, pulse voltage detection and peak value extraction were conducted using analog circuits, incorporating pulse peak detectors and pulse height analyzers. With the advancement of digital circuit technology, LiMCA II transitioned to employing digital signal processors for these tasks, showcasing the evolution towards digital circuitry. In commercial iterations of LiMCA products, irrespective of the signal processing module’s nature, the hardware devices exclusively handle pulse peak detection and signal extraction, relegating host computer software to the roles of data analysis and presentation. However, the surge in software technology capabilities in recent times has rendered host computer software fully equipped to execute peak detection and extraction operations. For this investigation, the C# programming language, within the Windows Presentation Framework (WPF) and utilizing the Visual Studio 2019 editor, facilitated the independent development of host computer software designed for the real-time online monitoring of inclusion distributions. This software extends beyond mere data analysis and visualization to encompass the extraction of pulse peak signals directly from the raw signal. As illustrated in Figure 5, the software’s user interface is compartmentalized into three distinct sections: a data presentation section, a log output section, and a status section. The data presentation section is engineered to exhibit various forms of dynamic data, refreshing this information in real time. The log output section serves to chronicle user interactions and instrument settings, whereas the status section primarily functions to exhibit and adjust instrument status parameters.

**Figure 6:**
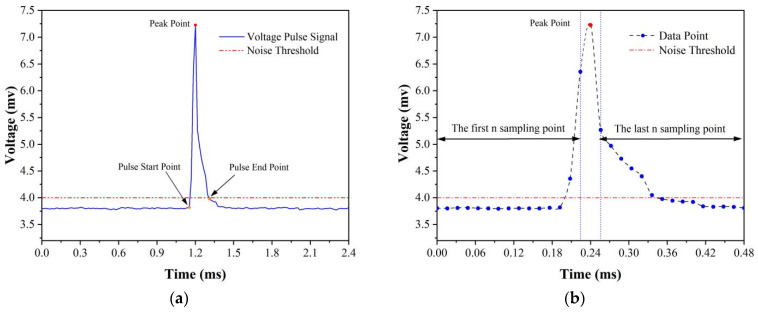
Signal process principle of peak detection algorithm: (**a**) principle of the peak detection algorithm in LiMCA products; (**b**) principle of the peak algorithm utilized in this work.

Beyond the fundamental functionalities of the host computer’s front-end described previously, the system’s back-end is capable of extracting the pulse peak from the raw signal and leveraging the pulse–particle size conversion equation to transform the voltage signal into the size of inclusions. To guarantee data timeliness, this study implemented an innovative peak detection algorithm that, in comparison to algorithms employed in commercial LiMCA products, processes identical data volumes more swiftly, thus ensuring the immediate visualization of calculated data on the software’s front-end. The operational principle of the peak detection algorithm in LiMCA products, depicted in Figure 6a, is initiated by setting a minimum value for the signal pulse peak, known as the noise threshold. This threshold mirrors the testing site’s conditions and stipulates the smallest detectable inclusion size under such circumstances. Sampling commences when the voltage signal first surpasses the noise threshold, marking the pulse’s onset, and concludes once the signal dips below this threshold, indicating the pulse’s termination. The segment of the voltage signal between these two points constitutes the captured inclusion pulse signal. This approach necessitates sequential comparison of each voltage signal value against the noise threshold, resulting in significant time complexity and, at high signal sampling rates, challenges in maintaining data’s real-time performance. The peak detection algorithm introduced in this research, illustrated in Figure 6b, evaluates each voltage signal against the values at n time points both prior and subsequent. A signal is identified as a pulse peak if it exceeds the adjacent 2n voltage signal values, with n, an algorithmic parameter, determined by the pulse signal’s transient duration and sampling rate. The identified pulse peak is then assessed against the noise threshold to isolate the inclusion pulse signal. Unlike traditional methods, this algorithm requires only comparisons to 2n points, significantly reducing time complexity.

## 4. Experiments and Analysis of Results

### 4.1. Analysis of the System’s Online Monitoring Function

This study and commercial LiMCA products employ two distinct peak detection algorithms—Algorithm A (utilized in this research) and Algorithm B (utilized in commercial LiMCA products)—to conduct peak extraction tests on a data segment. This dataset has a sampling rate of 62.5 kHz, encompassing 2,900,000 sampling points and includes 192 inclusion signal pulses. Table 1 presents the test outcomes, where “false detections” denote instances of incorrect identification of non-inclusion signals as inclusion pulses, and “missed detections” represent inclusion pulses the algorithms failed to recognize. The evaluation procedure entailed using both algorithms to identify pulse signals within the data segment, followed by manual verification to ascertain the counts of false detections and missed detections. An increase in either metric was recorded upon discovering an unrecognized inclusion pulse or a falsely marked non-inclusion pulse. Despite the similarity in missed detection and false detection rates between the two algorithms, indicating comparable precision in identifying pulse peaks, Algorithm B required 3.9 times more time for peak detection than Algorithm A. Given the critical need for real-time data in instruments and devices, the novel peak detection algorithm (Algorithm A) offers superior advantages in detection speed.

Samples of aluminum melt from identical batches and measurement locations were obtained using LiMCA CM instruments (Asea Brown Boveri, Zurich, Switzerland) at industrial sites and subsequently subjected to continuous measurement via a device integrated with an online inclusion monitoring system, with experimental data recorded across 15 time intervals. To conduct the measurements, the crucible containing the aluminum melt sample was positioned inside a muffle furnace (model ZK-5GY12TP) (ZHONGKEBEIYI, Beijing, China) and heated to a set temperature of 800 °C. Simultaneously, the sampling tube, featuring a 500 μm diameter orifice, was attached to the measurement probe. Upon reaching the target temperature, the probe was lowered until the sampling tube’s orifice was submerged in the molten metal, initiating the measurement process with a consistent current of 50 A.

**Figure 7:**
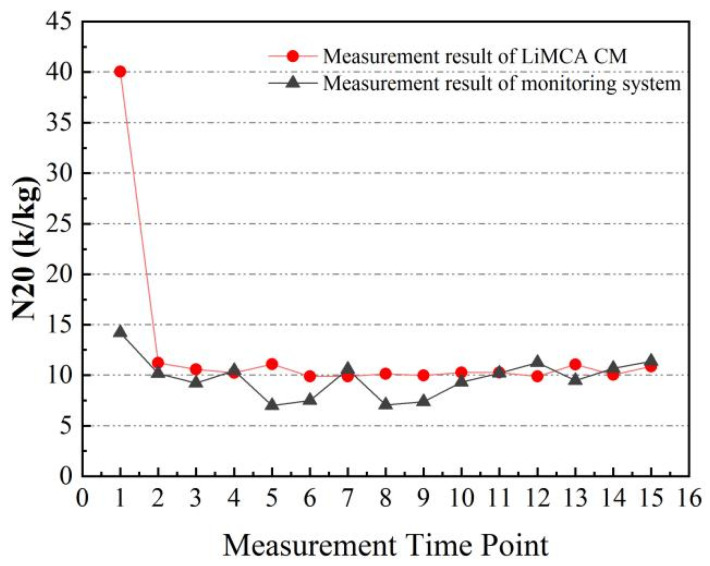
Comparison of the test results of the self-developed experimental measuring device and LiMCA CM.

The outcomes depicted in Figure 7 reveal that, after an initial period, the measurement results from both the monitoring device and the LiMCA product stabilized around a constant value. The early measurement data exhibited abnormal fluctuations due to significant current variations as the ESZ formed its initial path. However, with time, the current and, subsequently, the N20 data, indicating the number of inclusions per kilogram of aluminum melt, achieved stability. In industrial contexts, the mean value of stable N20 data serves as an indicator of the sample’s purity. In this experiment, the host computer interface updated real-time data at a 10 Hz frequency, presenting an average N20 value of 10.37 k/kg for the LiMCA products, compared to 9.72 k/kg measured by the monitoring device, indicating a reasonable discrepancy of approximately 6.3% when considering the variability introduced by the sampling process and the differing conditions between casting site and laboratory environments.

The initial variability in measurement data could stem from significant current fluctuations as the ESZ first establishes a pathway. With time, as the current becomes stable, the data likewise stabilize. Another potential cause for the early data fluctuations is the adherence of aluminum oxide film to the sampling orifice’s surface upon initial immersion in the aluminum melt. Continuous pumping may cause the oxide film to lodge within the orifice. However, over time, the constant flow of the liquid metal across the orifice can dislodge and remove the oxide film, making the orifice smoother and allowing the measurement data to stabilize.

**Figure 8:**
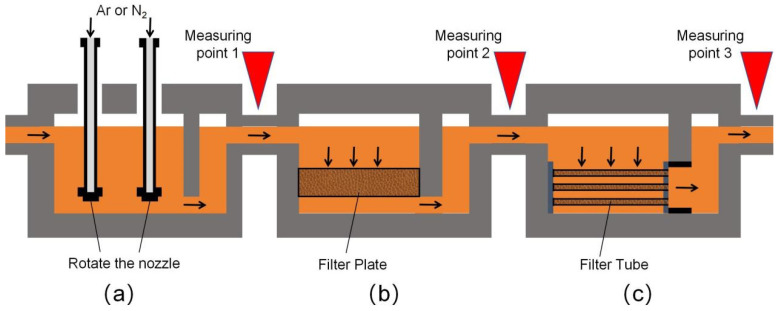
Schematic diagram of the filtering process, (The black arrows in the diagram show the direction of molten metal flow). (**a**) SNIF (spinning nozzle inert flotation); (**b**) CFF (ceramic foam filter); (**c**) MCF (metallics cartridge filter).

The evaluation of the filtration process in aluminum alloy casting utilized a monitoring device equipped with an online system for detecting inclusions. Key measurement points, as depicted in Figure 8, include stages post-SNIF, post-CFF, and post-MCF, with the outcomes also illustrated in Figure 8. In this experiment, a sampling tube with a 500 μm orifice was used, maintaining a constant current of 50A. The minimum detectable inclusion size, initially set at 20 μm for LiMCA products, was reduced to 16 μm. Equation (1) suggests that employing a sampling tube with a smaller diameter of 300 μm can decrease this detection threshold further, which was verified by extending the lower limit of detection to 10 μm. However, it was observed that sampling tubes with a 300 μm orifice are more susceptible to clogging by inclusions, likely due to inclusion clusters or oxide films in the aluminum melt obstructing the orifice.

### 4.2. Measurement Results and Analysis of Inclusions in Filtration Processes

**Figure 9:**
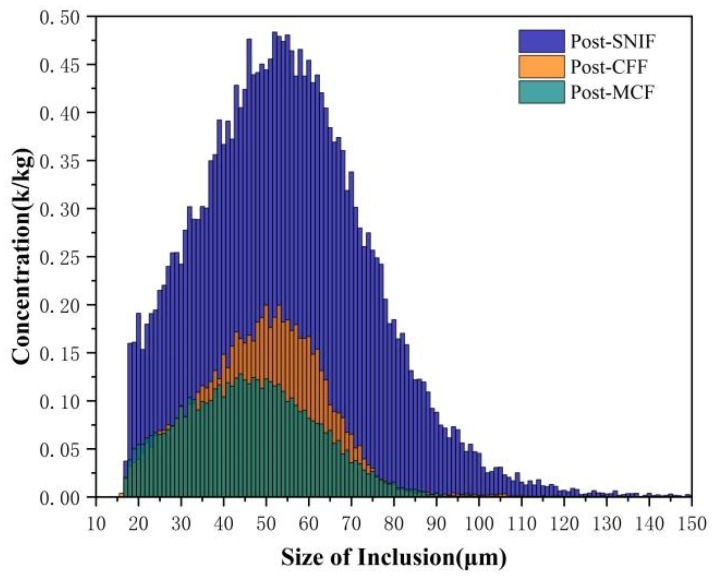
The average concentration distribution of inclusions of each size post-SNIF, post-CFF, and post-MCF.

This study analyzed three key stages, post-SNIF, -CFF, and -MCF, with Figure 9 presenting a comparative analysis of the average concentrations of different-sized inclusions at these stages. The experimental data revealed that the largest inclusions in the aluminum melt post-SNIF measured up to 150 μm, with inclusions measuring 52 μm exhibiting the highest concentration, recorded at 0.48 k/kg. Following the CFF and MCF stages, the maximum inclusion sizes were observed to decrease to 119 μm and 100 μm, respectively. Specifically, post-CFF, the most prevalent inclusion size was 50 μm, with a concentration of 0.21 k/kg; post-MCF, the most common size was 44 μm, noted at a concentration of 0.13 k/kg. These findings indicate a significant reduction in both the maximum size of inclusions and their overall concentrations following CFF, highlighting its effectiveness in filtering inclusions of all sizes.

Table 2 presents the results of inclusion measurements following SNIF, CFF, and MCF processes. The N20–N80 inclusion counts post-SNIF were 23.22 k/kg, 20.97 k/kg, 17.87 k/kg, 13.68 k/kg, 9.06 k/kg, 5.11 k/kg, and 2.52 k/kg, respectively, with inclusion sizes ranging from 17 to 150 μm. The majority of inclusions after SNIF, accounting for between 79% and 90%, fell within the 30 to 80 μm range, whereas inclusions larger than 80 μm comprised only 5%, aligning with the observed outcomes of the refining process.

Post-CFF, the average N20–N80 values significantly decreased to 6.75 k/kg, 6.03k/kg, 4.89 k/kg, 3.20 k/kg, 1.42 k/kg, 0.38 k/kg, and 0.09 k/kg, respectively. The distribution of inclusions predominantly lay within the 20 to 70 μm range, making up 94% of the total, with less than 1% exceeding 90 μm. The inclusion concentration was reduced by 71% after CFF, underscoring its substantial filtration efficacy.

Following MCF treatment, the average N20–N80 values were further reduced to 4.9 k/kg, 4.15 k/kg, 3.14 k/kg, 1.93 k/kg, 0.91 k/kg, 0.3 k/kg, and 0.07 k/kg, respectively. The inclusion range narrowed to 30 to 60 μm, with a 79% decrease in overall inclusion concentration, highlighting the combined effectiveness of CFF and MCF in inclusion removal, particularly for those above 60 μm.

Table 3 evaluates the filtration efficiency at three critical measurement points for inclusions measuring 10 μm. CFF served as the initial filtration step post-SNIF, targeting large inclusions and playing a crucial role in inclusion management. MCF acted as a secondary filtration stage, enhancing cleanliness by fine-filtering remaining inclusions. The combined CFF and MCF process was pivotal in ensuring aluminum melt purity. The data from Table 3 indicate that CFF achieved approximately 60% efficiency for inclusions under 60 μm, rising to 68% for 20–29 μm inclusions. This efficiency increased with inclusion size, reaching 88% for 70–79 μm inclusions and 97% for those above 80 μm, evidencing a 35% improvement over smaller inclusion filtration.

**Figure 10:**
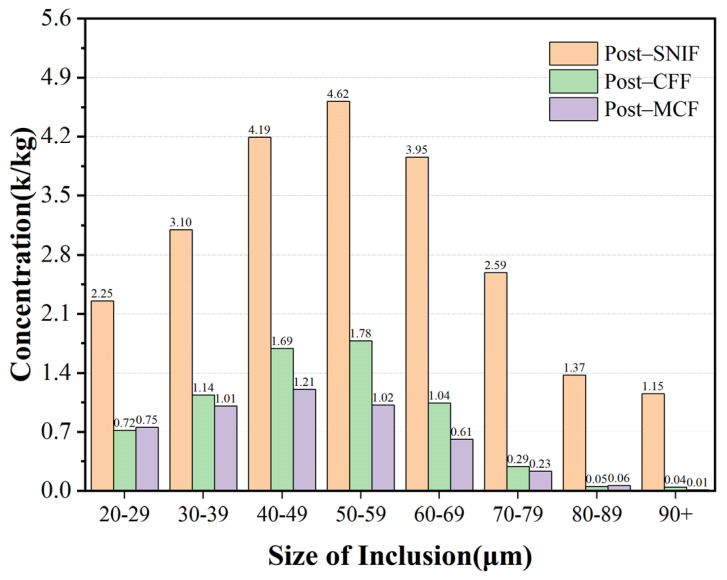
Average concentration of a set of inclusions at 10 μm after SNIF, CFF, and MCF.

MCF acted as a secondary filtration mechanism, significantly enhancing the removal of inclusions. Specifically, it achieved a filtration efficiency of over 70% for inclusions smaller than 60 μm, with an impressive 77% efficiency for inclusions measuring 50–59 μm. The application of MCF to inclusions ranging from 30 to 79 μm markedly improved filtration efficiency, contributing to an overall capacity increase of about 10%. As illustrated in Figure 10, the combined CFF + MCF filtration process dramatically reduced inclusions larger than 90 μm from 1.15 k/kg post-SNIF to 0.01 k/kg, achieving a filtration efficiency exceeding 99%. This effectively eliminated nearly all inclusions larger than 90 μm. However, the factory’s filtering equipment’s performance on smaller-sized inclusions was less optimal. For inclusions measuring 20–29 μm, the filtration efficiency stood at approximately 66%, which was lower than the over 90% efficiency observed for inclusions larger than 70 μm.

The primary filtration through CFF, which targeted inclusions between 20 to 60 μm, only reached about 60% efficiency. Consequently, a significant number of inclusions bypassed this stage and entered MCF. The MCF filter tubes’ large specific surface area and high adsorption capacity for large-sized inclusions led to the formation of a “cake layer” on their surfaces. This layer obstructed the flow, and the increased melt weight and flow rate caused the melt to rush through the channels more swiftly, potentially dislodging small inclusions and allowing some to bypass the filtration layer. This phenomenon may have resulted in a slight increase in inclusion concentrations, aligning with observed on-site process dynamics.

**Figure 11:**
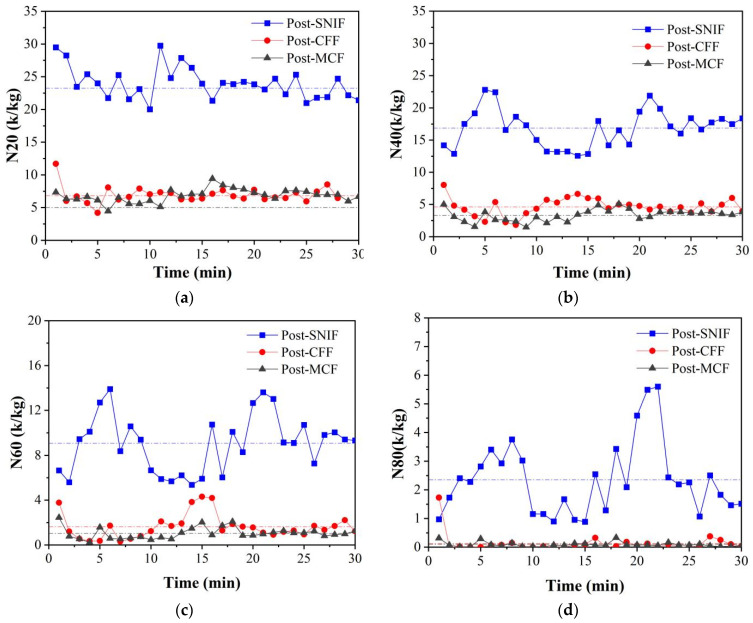
Changes in N20, N40, N60, and N80 counts after SNIF, CFF, and MCF with measurement time: (**a**) N20 data; (**b**) N40 data; (**c**) N60 data; (**d**) N80 data.

Throughout the measurement period, changes in suction pressure may have induced fluctuations in the velocity of inclusions passing through the ESZ. However, within the effective measurement timeframe, the N20–N80 metrics exhibited fluctuations below 5 k/kg, demonstrating minimal variability. With prolonged measurement, the N20–N60 metrics stabilized at approximately 1 k/kg, signaling a stabilization of conditions within the ESZ, as depicted in Figure 11.

To intuitively quantify the impurity removal efficacy of the combined primary and secondary filtration, the temporal changes in the N20, N40, N60, and N80 metrics were monitored. As shown in Figure 11, the inclusion metrics for aluminum melt post-SNIF, -CFF, and -MCF remained around the average value, with fluctuations within a 5 k/kg range, aligning with LiMCA product standards.

The aluminum melt’s inclusion distribution was notably uneven and aggregated due to melting, standing, refining, and other processes, leading to significant fluctuations in inclusion concentration post-SNIF. This was evidenced by variations typically under 10 k/kg, reflecting the dynamics of the refining and degassing processes, where some inclusions rose with gases while others remained scattered within the melt. The N80 data’s fluctuation post-SNIF, limited to about 5 k/kg, underscored the pre-filtration processes’ efficacy in removing larger inclusions (over 80 μm), highlighting their contribution to impurity mitigation.

The average N20 inclusion, counted at three measurement points—post-SNIF, post-CFF, and post-MCF—were 33.62 k/kg, 6.82 k/kg, and 5.01 k/kg, respectively. Following the initial CFF filtration stage, the N20 inclusion count decreased by 79.71%. Subsequent to the combined CFF + MCF filtration stages, this count further reduced by 85.1%, demonstrating that the filtration process could remove over 80% of inclusions in the aluminum melt. Similarly, the average N40 inclusion counts were 16.86 k/kg, 4.65 k/kg, and 3.33 k/kg, respectively, with a 72.36% reduction after CFF treatment and an 80.25% reduction following MCF treatment, indicating a consistent decrease in N40, N60, and N80 inclusion counts after both CFF and MCF treatments.

Post-SNIF, the concentration of inclusions larger than 80 μm stood at 2.34 k/kg, which significantly decreased after filtration, reaching just 0.13 k/kg post-CFF and 0.09 k/kg post-MCF. This substantial reduction nearly eliminated all inclusions larger than 80 μm. The consistent downward trend in the N40, N60, and N80 data after both primary and secondary filtering stages suggested a regular pattern, where larger-sized inclusions were more effectively captured by the filters, highlighting the enhanced filtering efficiency for larger inclusions.

## 5. Conclusions

This study aimed to enhance the detection efficiency of inclusions in liquid aluminum by refining the signal acquisition module and peak detection functionality within an online monitoring system framework. A novel signal acquisition module was developed, incorporating a multiplexer to allow real-time adjustments of signal gain and filter bandpass. This advancement elevated the detection threshold for inclusions to 16 μm, improving the monitoring device’s stability across various environments and broadening its applicability to assessing the cleanliness of high-temperature liquid metals beyond aluminum.

This research represents the inaugural effort to incorporate signal pulse peak extraction functionalities within host computer software. Unlike the traditional approach of utilizing hardware devices for pulse peak extraction in LiMCA products, the signal processing module developed here streamlines the digital signal processing architecture and lowers the cost associated with pulse peak extraction. Moreover, the peak detection algorithm introduced in this study markedly enhances detection efficiency over LiMCA products, meeting the real-time demands of the signal processing module for both peak detection and data visualization.

Building upon the development of an online monitoring system for inclusions, a specialized monitoring device was engineered to assess the aluminum melt processed through SNIF, CFF, and MCF treatments within the aluminum alloy casting workflow. Utilization of this proprietary device revealed that pre-SNIF inclusion sizes ranged from 16 to 150 μm, which were reduced to a span of 16–80 μm post-MCF. Notably, the inclusion concentration peaked at 52 μm (0.48 k/kg) prior to SNIF, decreasing to 50 μm (0.21 k/kg) post-CFF, and further to 44 μm (0.13 k/kg) following MCF. The filtration efficiencies for N20, N40, N60, and N80 inclusion sizes after applying CFF + MCF dual filtration were observed to be 85.10%, 80.25%, 88.51%, and 96.15%, respectively, indicating that filtration was more effective for larger inclusions and that the combined CFF + MCF filtration significantly outperformed the single CFF approach.

Employing a sampling tube with a 500 μm orifice and maintaining a constant current of 50 A, the online monitoring system was adept at detecting inclusions larger than 16 μm, aligning with the bulk of metallurgical industry standards. Additionally, the system’s lower detection limit could be refined in two ways: firstly, by increasing the constant current or decreasing the sampling tube’s orifice to reduce the lower limit; secondly, through the optimization of circuit design and signal processing techniques, which can lower the background noise and enhance the signal-to-noise ratio, thereby diminishing the minimum size of detectable inclusions.

The monitoring device, integrated with an online system for inclusion detection, facilitates real-time, online, and in situ measurement of aluminum melt. This enhances detection precision and expedites the process, enabling a detailed analysis of how filtration affects inclusion distribution and variability. Consequently, it lays the groundwork for refining filtration methods and managing inclusion quality. Additionally, it opens avenues for the online detection of inclusions in molten materials like aluminum, zinc, steel, and beyond, offering innovative perspectives for industry applications.

## Figures and Tables

**Table 1:** Comparison of the performance of the two peak algorithms.

Algorithm	Number of False Detections	Number of Missed Detections	False Detection Rate%	Miss Detection Rate%	Running Time/ms	Number of Peaks
A	5	3	2.60	1.56	96	192
B	4	3	2.08	1.56	374	

**Table 2:** Measurement results of inclusions after SNIF, CFF, and MCF processes.

Measure Position	N20 /k/kg	N30 /k/kg	N40 /k/kg	N50 /k/kg	N60 /k/kg	N70 /k/kg	N80 /k/kg
Post-SNIF	23.22	20.97	17.87	13.68	9.06	5.11	2.52
Post-CFF	6.75	6.03	4.89	3.2	1.42	0.38	0.09
Post-MCF	4.9	4.15	3.14	1.93	0.91	0.3	0.07

**Table 3:** Average filtration efficiency of inclusions in groups of 10μm.

Inclusion Size/(μm)	Post-SNIF Concentration of Inclusions/(k/kg)	Post-CFF Concentration of Inclusions/(k/kg)	Post-MCF Concentration of Inclusions/(k/kg)	CFF Filter Efficiency/%	CFF + MCF Filter Efficiency/%
20–29	2.25	0.72	0.75	68.00	66.66
30–39	3.10	1.14	1.01	63.23	67.41
40–49	4.19	1.69	1.21	59.67	71.12
50–59	4.62	1.78	1.02	61.47	77.92
60–69	3.95	1.04	0.61	73.67	85.55
70–79	2.59	0.29	0.23	88.80	91.11
80–89	1.37	0.05	0.06	96.35	95.62
Over 90	1.15	0.04	0.01	96.52	99.13

## Data Availability

Data are contained within the article.
